# Comparative analysis of the tumor microbiome, molecular profiles, and immune cell abundances by HPV status in mucosal head and neck cancers and their impact on survival

**DOI:** 10.1080/15384047.2024.2350249

**Published:** 2024-05-09

**Authors:** Rituraj Upadhyay, Aastha Dhakal, Caroline Wheeler, Rebecca Hoyd, Malvenderjit Jagjit Singh, Vidhya Karivedu, Priyanka Bhateja, Marcelo Bonomi, Sasha Valentin, Mauricio E. Gamez, David J. Konieczkowski, Sujith Baliga, John C. Grecula, Dukagjin M. Blakaj, Emile Gogineni, Darrion L. Mitchell, Nicholas C. Denko, Daniel Spakowicz, Sachin R. Jhawar

**Affiliations:** a Department of Radiation Oncology, The Ohio State University Comprehensive Cancer Center, Columbus, OH, USA; b The Ohio State University College of Medicine, Columbus, OH, USA; c Division of Medical Oncology, The Ohio State University Comprehensive Cancer Center, Columbus, OH, USA; d Department of Dentistry, The Ohio State University Wexner Medical Center, Columbus, OH, USA; e Department of Radiation Oncology, Mayo Clinic, Rochester, MN, USA; f Pelotonia Institute for Immuno-Oncology, Columbus, OH, USA

**Keywords:** Tumor microbiome, molecular profiles, immune cell abundances, HPV, HNSCC, microbiome

## Abstract

Head and Neck Squamous Cell Carcinoma (HNSCC) comprises a diverse group of tumors with variable treatment response and prognosis. The tumor microenvironment (TME), which includes microbiome and immune cells, can impact outcomes. Here, we sought to relate the presence of specific microbes, gene expression, and tumor immune infiltration using tumor transcriptomics from The Cancer Genome Atlas (TCGA) and associate these with overall survival (OS). RNA sequencing (RNAseq) from HNSCC tumors in TCGA was processed through the exogenous sequences in tumors and immune cells (exotic) pipeline to identify and quantify low-abundance microbes. The detection of the Papillomaviridae family of viruses assessed HPV status. All statistical analyses were performed using R. A total of 499 RNAseq samples from TCGA were analyzed. HPV was detected in 111 samples (22%), most commonly Alphapapillomavirus 9 (90.1%). The presence of Alphapapillomavirus 9 was associated with improved OS [HR = 0.60 (95%CI: 0.40–0.89, *p* = .01)]. Among other microbes, *Yersinia pseudotuberculosis* was associated with the worst survival (HR = 3.88; *p* = .008), while *Pseudomonas viridiflava* had the best survival (HR = 0.05; *p* = .036). Microbial species found more abundant in HPV- tumors included several gram-negative anaerobes. HPV- tumors had a significantly higher abundance of M0 (*p* < .001) and M2 macrophages (*p* = .035), while HPV+ tumors had more T regulatory cells (*p* < .001) and CD8+ T-cells (*p* < .001). We identified microbes in HNSCC tumor samples significantly associated with survival. A greater abundance of certain anaerobic microbes was seen in HPV tumors and pro-tumorigenic macrophages. These findings suggest that TME can be used to predict patient outcomes and may help identify mechanisms of resistance to systemic therapies.

## Introduction

Head and Neck Squamous Cell Carcinomas (HNSCC) comprise a diverse group of tumors. In 2023, there were 54,540 new cases and 11,580 deaths due to HNSCC.^1^ Current prognostic factors primarily rely on clinical features such as patient age, stage, disease site, performance status, smoking history, and presence of human papilloma virus (HPV).^2^ The role of HPV in pathogenesis of HNSCC has been well described, and oropharyngeal squamous cell carcinomas (OPSCC) in particular, are divided into subtypes based on the presence of HPV.^3,4^ While the high-risk HPV produces oncoproteins E6 and E7 that target TP53 and retinoblastoma protein respectively, leading to increased proliferation,^5^ the pathogenesis of HPV-negative HNSCC has not been as well elucidated. HPV-positive OPSCC are known to have better treatment response and patient outcomes compared to HPV-negative tumors,^6^ however, the molecular basis of the differences between the two subtypes is poorly understood.

Traditional clinical and molecular prognostic factors offer valuable insight into the heterogeneous natural history and treatment response, but fail to explain the full spectrum of observed variability in the clinical behavior of HNSCCs. In this setting, developing modern prognostic and predictive markers, especially with the increased use of immunotherapy in several cancers, is warranted. The advent of immunotherapy has driven studies toward variables that affect immune system infiltration and thereby can predict treatment response. However, these efforts have uncovered a role of the immune system in the response to other treatment modalities, as well, including radiation and chemotherapy. The tumor microenvironment (TME), a multicellular region surrounding the tumor, comprised of microbes and immune cells, has been observed to impact treatment response and prognosis.^7^ A major component of the TME is tumor-associated macrophages (TAM) which are involved in promoting cancer invasion and migration.^8^ The proinflammatory cytokines like Tumor Necrosis Factor alpha (TNF-α) and Interferon-gamma (IFN-γ) give rise to M2 macrophages, and these macrophages produce anti-inflammatory responses (IL-10 and TGF-beta, regulatory T cells) which result in an immunosuppressive effect, allowing the cancer cells to escape Th1 mediated anti-tumor immunity.^8,9^

The impact of immune cell composition on the TME has been previously explored. In HNSCC, as well as gastric and urogenital cancer, higher numbers of TAM are associated with poor outcomes.^10^ Studies have also found that an increase in circulating T regulatory (Treg) cells is associated with poor prognosis in HNSCC patients, while infiltration of CD8+ T-cells in the tumor is associated with better local control.^11,12,13^ How the tumor microbiome affects these associations is under-explored.

In this study, we analyzed HNSCC bulk RNA sequencing data to evaluate the association of specific microbes in the TME with survival. We also explored the differential expression of specific genes and immune cells in HPV-positive and HPV-negative HNSCC, with a goal to develop predictive markers and future therapy targets that can improve treatment responses and, eventually, patient outcomes.

## Methods

We analyzed gene expression in the tumor samples from The Cancer Genome Atlas (TCGA) dataset to identify microbes and immune cells in HNSCC samples. TCGA RNA sequencing samples (RNAseq) were processed through the exogenous sequencing in tumors and immune cells (ExoTIC) pipeline to identify and count low abundance microbes, including bacteria, fungi, viruses, archaea, and select other eukaryotes; filter contaminants; and normalize expression values.^14^

The detection of the Papillomaviridae family of viruses assessed HPV status. Samples were classified as HPV-positive if >5 reads mapped to genomes in the Papillomaviridae family, including Alphapapillomavirus 1 (no. of patients, *n* = 2); 4 (*n* = 1); 5 (*n* = 4); 7 (*n* = 14); 9 (*n* = 100); 11 (*n* = 7); 12 (*n* = 6); 13 (*n* = 2); Betapapillomavirus 1 (*n* = 2); 4 (*n* = 1); Gammapapillomavirus 5 (*n* = 1); Dyokappapapillomavirus 4 (*n* = 1); Iotapapillomavirus 2 (*n* = 1); and Human papillomavirus type 85 (*n* = 1). Presence of HPV based on p16 or HPV-DNA status was not available in the TCGA database.

Available clinical data from TCGA was also extracted to compare overall survival (OS) and control for confounding variables. All continuous data were represented as mean and standard deviation (SD). Cox proportional hazards regression was used to identify the association of microbes with OS. The multivariate Cox hazard regression model controlled for age, stage, and smoking status. An adjusted p-value of <.05 was considered significant.^15^

Differential expression of microbes between HPV-positive and HPV-negative tumors was plotted using negative log (base two) fold change. Immune cell abundances were estimated by deconvolution using high-quality immune cell reference datasets. Difference in immune cell abundances were evaluated by Kruskal–Wallis test. Models were constructed using the {stat} package in R.^16^ Code to reproduce all analyses and figures is available at https://github.com/ [[remainder of the URL deleted because it contains identifiable author information]].

## Results

A total of 499 RNAseq samples from TCGA were analyzed (Table 1). Most patients were male (73.3%), and the majority had AJCC 7th edition stage IV HNSCC (53.9%). The number of patients with primary tumors in the oral cavity, oropharynx, hypopharynx, and larynx was 303 (60.7%), 75 (15.0%), 9 (1.8%), and 111 (22.2%), respectively. Overall, HPV was present in 111 patients (22%), of which the most common type was Alphapapillomavirus 9 seen in 100 patient samples (90.1%). Alphapapillomavirus 9 is a species of the Papillomaviridae family that includes several HPV strains including HPV 16, 31, 33, 35, 52, 58 and 67, while the other high-risk strain, HPV 18 is included in Alphapapillomavirus 7 species (*n* = 14).

**Table 1.** The demographic profile of the RNAseq samples from the Cancer Genome Atlas.

Patient Characteristics	
N	499
Age [(mean (SD)]	61.55 (11.92)
Gender (%)	
Male	366 (73.3)
Female	133 (26.7)
Clinical Stage (%)	
Stage I	19 (3.8)
Stage II	95 (19.0)
Stage III	102 (20.4)
Stage IV	269 (53.9)
Unknown	14 (2.8)
Primary Site (%)	
Oral cavity	303 (60.7)
Oropharynx	75 (15.0)
Hypopharynx	9 (1.8)
Larynx	111 (22.2)
Other	1 (0.2)
Vital Status at last follow-up Deceased (%)	217 (43.5)
HPV-positive (%)	111 (22.2)

**Figure 1.**
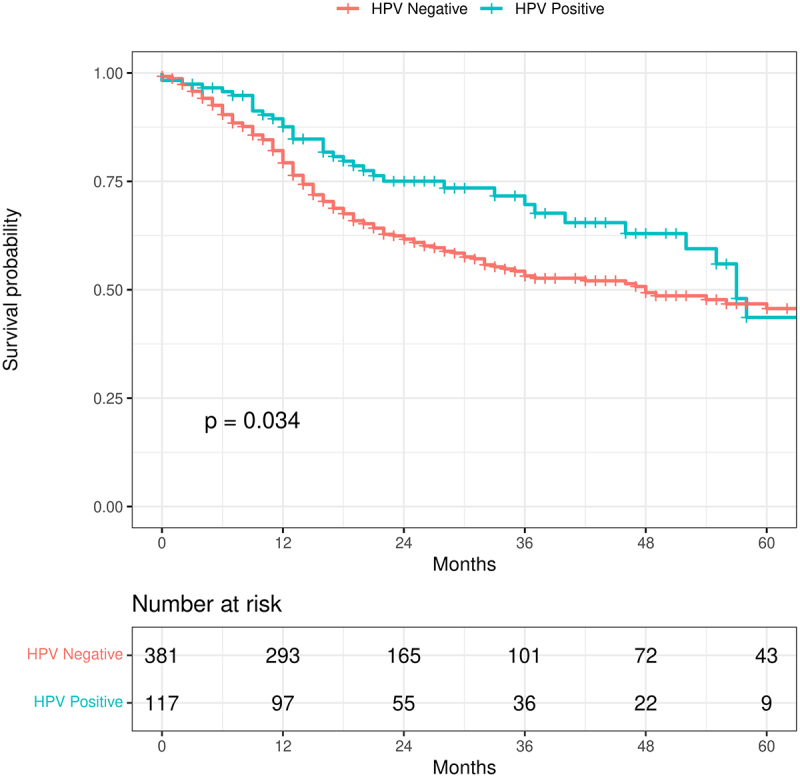
Kaplan-Meier curve showing the difference in survival between the HPV-positive and HPV-negative tumors. HPV-positive tumors aggregate several papilloma virus strains. Patients with HPV-positive HNSCC have better overall survival. This curve does not control for covariates.

The ExoTIC pipeline identified 5963 microbes including bacteria, viruses, and fungi enriched in the tumor samples. To test whether our RNA-seq-based observation of HPV status yielded consistent results with established clinical outcomes, we first used HPV status to stratify OS. As expected, patients who had HPV-positive tumors had improved overall survival (Figure 1, *p* = .034). Furthermore, we sought to ensure that this association remained true after controlling for covariates with an established effect on OS. After controlling for stage, smoking status, and age, HPV remained a significant predictor of OS. This observation was further specified to genome resolution, and the presence of Alphapapillomavirus 9 (the most common species of HPV in the sample) was associated with significantly improved OS [Hazard ratio (HR) for death = 0.60, 95% CI: 0.40–0.89, *p* = .011], while controlling for stage, smoking status, and age (Supplementary Figure S1).

**Figure 2.**
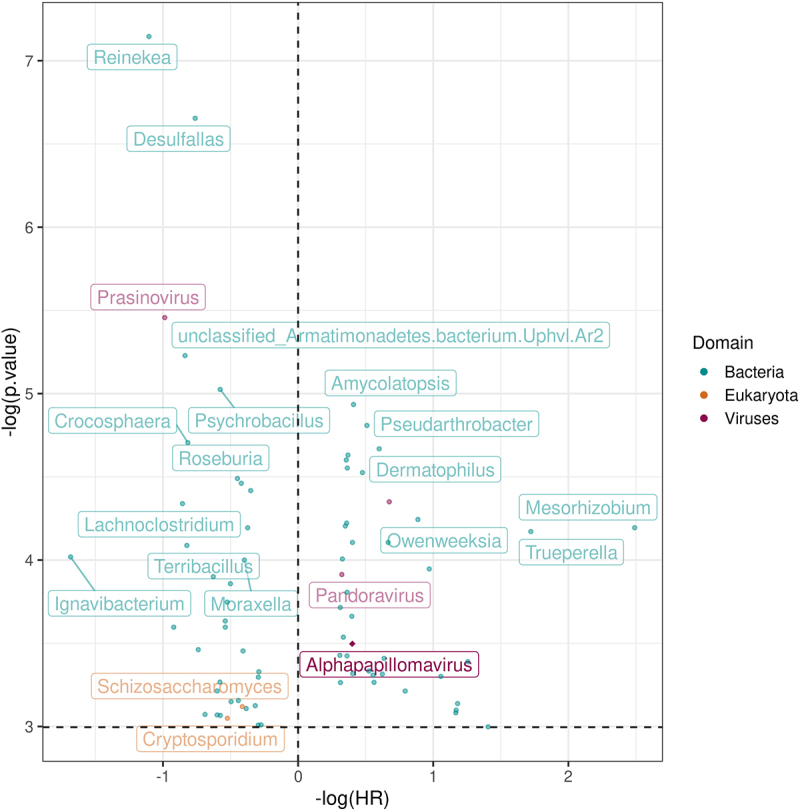
Hazard ratio (x-axis) and p-values (y-axis) generated from the cox-hazard proportional model that analyzed the overall survival with the presence of each microbe, controlling for stage, smoking status, and age. The diamond represents the alphapapillomavirus 9, a strain of HPV.

We then tested the association of microbes with survival with genera found in at least 10 samples. Of these, 76 significantly associated with OS, with largest hazard ratio associated with the presence of the genus Reinekea (HR = 3.02, 95% CI: 1.58–5.74, adjusted *p* = .046) (Figure 2, complete list in Supplementary Table S1).

**Figure 3.**
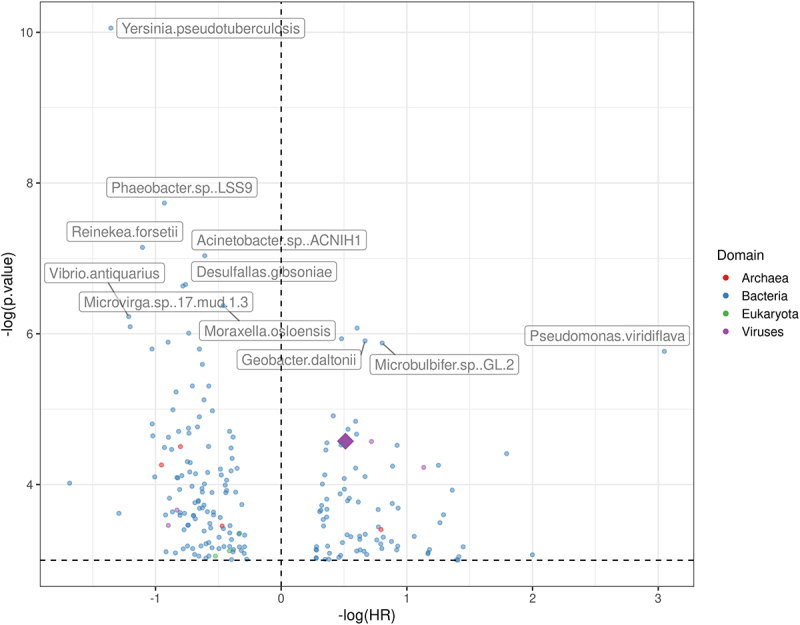
Hazard ratio (x-axis) and p-values (y-axis) from the cox-hazard proportional model analyzing the overall survival with the presence of each microbe, after excluding microbes present in less than 10 samples.

Further examining species-level identities, 208 microbes were significantly associated with OS after applying the same filter (present in >10 samples). The microbe whose presence was associated with highest hazard ratio was Yersinia pseudotuberculosis (HR = 3.88, 95% CI: 2.02–7.42, adjusted *p* = .008), while the microbe associated with lowest was Pseudomonas viridiflava (HR = 0.05, 95% CI: 0.01–0.36, adjusted *p* = .036) (Figure 3, complete list in Supplementary Table S2).

**Figure 4.**
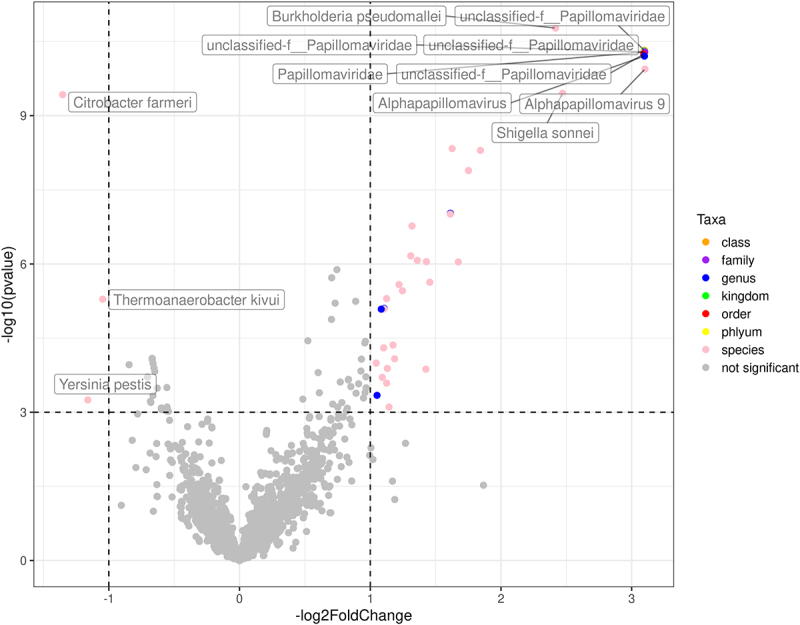
Differentially expressed microbes by HPV-status. The x-axis shows HPV-negative (left, negative -log2 fold change) and HPV-positive (right, positive -log2fold change), and the y-axis is the negative log of p-value for the microbes associated with each type of tumor.

We performed comparative analysis to evaluate microbes and genes associated with HPV-positive and HPV-negative tumors. Figure 4 shows the microbes which were found to be distinct in HPV-negative and HPV-positive tumors. Microbial species found in more abundance in HPV-negative tumors included *Citrobacter farmeri* (gram negative facultative anaerobe), *Thermoanaerobacter kivui* (gram negative anaerobe) and *Yersinia pestis* (gram negative facultative anaerobe). Several species not in the Papillomaviridae family seen in more abundance in HPV-positive tumors are listed in Figure 4 (complete list in Supplementary Table S3).

We also analyzed the molecular differences between HPV-positive and HPV-negative HNSCC. We found that the genes related to cellular processes like cellular transport and cell migration, and DNA repair were enriched in HPV-positive samples. In contrast, genes associated with proliferation (SAGE1) and transport (BPIFB2) were depleted in HPV-positive samples (Supplementary Figure S2, complete list in Supplementary Table S4). We used a gene-set enrichment analysis to identify the pathways overrepresented by the differentially expressed genes. Genes encoding cell cycle related targets of E2F transcription factors; genes involved in the G2/M checkpoint of cell cycle, genes important for mitotic spindle assembly, genes up-regulated during spermatogenesis, and genes involved in DNA repair were significantly enriched in HPV-positive tumors compared to HPV-negative tumors (Supplementary Figure S3, complete list in Supplementary Table S5).

**Figure 5.**
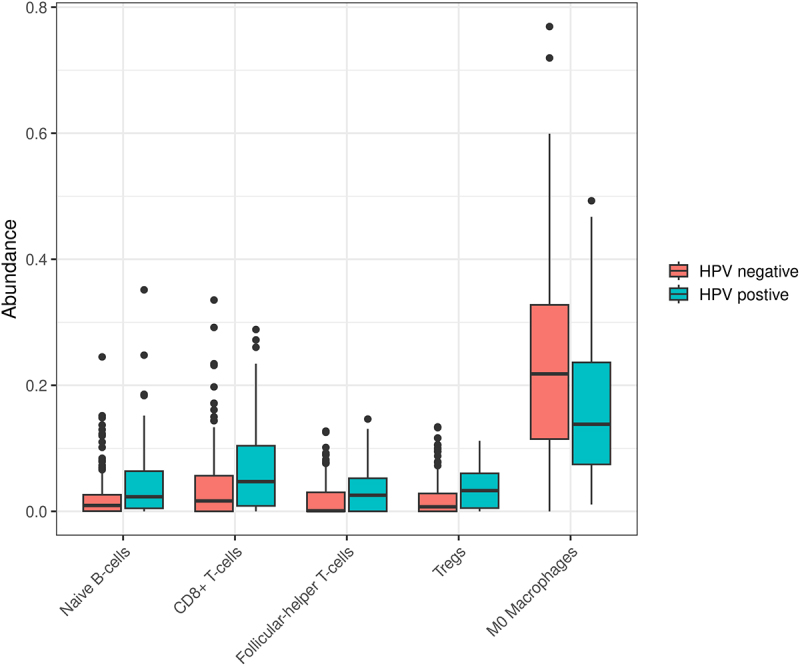
Relative abundance of pertinent immune cell types found in HPV-positive and negative tumors. CD8+ T cells, helper T cells, and naïve B cells were found to be more abundant in HPV-positive tumors. M0 macrophages were more abundant in the HPV-negative tumors.

We then analyzed the differences in the immune cell composition of HPV-positive and negative samples (Figure 5, complete list in Supplementary Table S6).

A total of 22 different immune cell types were evaluated. Difference in median abundances was calculated using Kruskal–Wallis test. HPV-negative tumors had a significantly higher number of M0 (*p* < .001) and M2 macrophages (*p* = .035) while in HPV-positive samples, there were significantly more T regulatory cells (*p* < .001), CD8+ T-cells (*p* < .001), naive B-cells (*p* = .001), and follicular-helper T-cells (*p* = .001). HPV-positive tumors were somewhat more enriched in M1 macrophages, but the difference was not significant (*p* = .561).

## Discussion

Our study identified several microbes significantly associated with overall survival after controlling for confounding factors. Our results confirm that the tumor HPV status is a strong prognostic factor for survival in HNSCC patients.^17^ Other microbes significantly associated with survival were *Pseudomonas viridiflava* with improved survival and *Yersinia pseudotuberculosis* with worse survival. We also observed that certain microbial species were more abundant in HPV-negative tumors, including *Citrobacter farmeri, Thermoanaerobacter kivui* and *Yersinia pestis* which are all gram-negative anaerobes. As bacteria and fungi in the TME have been shown to affect radiation response,^18^ knowing specific microbes associated with HPV status can allow us to consider manipulation of the microbiome as a therapeutic strategy to make tumors more responsive to radiation therapy. Another genus associated with poorer survival was *Reinekea spp*., which is a facultative anaerobe, and a gammaproteobacteria. Such lipopolysaccharide-containing gram-negative bacteria are known to increase inflammation through Toll-like receptor signaling. In addition, tumor gamma proteins have been found to degrade gemcitabine as well as some other anti-metabolite chemotherapeutic agents and may negatively affect response to systemic therapy.^19^

We also observed that genes associated with cellular proliferation and transport were depleted and genes involved in cell cycle were enriched in HPV-positive tumors. It has been previously reported that genes associated with cell cycle and epithelial mesenchymal transition pathways are affected the most in HNSCCs, with the dysregulation dependent on the anatomic site of the tumor.^20^ Dysregulation of epidermal growth factor receptor (EGFR) expression is associated with various solid tumors, and overexpression of EGFR and TGF-α is correlated with poor survival in patients in HNSCC.^21^ Understanding the genetic changes in between HPV-positive and HPV-negative HNSCC may allow us to find distinct altered pathways that can be targeted for therapy.

In recent years, immunotherapy has been a promising strategy for the treatment of several cancers by activating tumor-antigen-specific T-cell responses. Despite the fact that a durable response has been observed in a subset of patients, the majority of tumors show only a limited clinical effect, and many do not respond.^22^ To overcome immune tolerance, the tumor-induced immune-suppressive mechanisms have been focused upon in recent years, and multiple suppressor cell populations in the tumor microenvironment (TME) have been identified, among which the tumor-associated macrophages (TAMs) stand out as novel targets to enhance immunotherapy effects,^23^ with multiple drugs being developed in this space. TAMs can be divided into two subtypes: M1 (antitumor immunity) and M2 (immunosuppression and tumor immune escape by suppressing T-cell function). M2-macrophages not only fail to exercise antitumor activities but can promote tumor growth and favor metastasis, helping create a TME nourishing the tumor. The M1/M2 ratio therefore serves as a potential biomarker of response to immunotherapy. Higher levels of tumor-infiltrating M2 are significantly associated with shorter survival, whereas a higher ratio of M1 to pan-macrophages (%M1) shows positive correlation with longer overall survival.^24^ M2 macrophages also play a key role in the initiation of metastasis through the secretion of pro-angiogenic cytokines and growth factors.^25,26^ In fact, M2 macrophages have been found to be more resistant to radiation therapy in the context of glioblastomas than M1 macrophages.^27^

We found increased M0 and pro-tumorigenic M2 polarized macrophages in HPV-negative tumors. The increased abundance of M2 macrophages in the HNSCC could allow the tumors to avoid immunosurveillance,^9^ leading to a poor outcome. Studies have shown that irradiated tissues have tumor microenvironment that promotes the accumulation and M2 polarization of macrophages.^28^ In breast cancer, pretreatment to inhibit M2 differentiation of macrophages has shown increased response to radiation.^29^ High and low doses of irradiation can reprogram M1 macrophages toward M2-like macrophages due to an increase of avascular hypoxia, whereas unpolarized macrophages tend to shift toward M1-like macrophages after moderate doses of irradiation.^30–32^ However, the effects of irradiation on macrophage reprogramming are dose-dependent, and low-dose irradiation and proton irradiation have been reported to reprogram TAMs, orchestrating the recruitment of cytotoxic lymphocytes.^33,34^ In this context, reprogramming M2 TAMs toward a more pro-inflammatory M1 phenotype can serve as a potential mechanism to improve treatment response in HPV-negative HNSCC patients. Toll-like receptors (TLRs) are key players in M1 programming, and targeting of several TLRs has been evaluated in the past few years. Imiquimod, a TLR7 agonist, is an immunomodulator FDA-approved for topical-only use for squamous and basal cell carcinomas.^35^ Several other molecules including nanoparticles are in development to target M2 TAMs.^36,37^

In our study, HPV-positive tumors had significantly more T regulatory cells, CD8+ T-cells, and follicular-helper T-cells compared to HPV-negative tumors. For patients with HNSCC, infiltrating T cells are associated with good prognosis.^38^ The increase in T cells in the microenvironment we found could potentially contribute to the improved prognosis of HPV-positive HNSCC compared to HPV-negative. Increased infiltration with T regulatory cells has been shown to be associated with worse outcomes, and this may be a potential target for HPV-positive patients who do not respond to immunotherapy. Our study also revealed a significantly greater abundance of naïve B-cells in HPV-positive tumors relative to HPV-negative tumors. HPV-positive patients with higher tumor expression of B-cell markers have improved overall survival.^39^ This is consistent with other cancer types as well, suggesting another potential biomarker that can direct therapeutic decisions.^40^

There are several limitations to our study, including the analysis being limited to the TCGA HNSCC datasets. Another limitation is the retrospective design using the heterogenous population available, and due to limited clinical data in the TCGA, we were unable to investigate the association of specific treatment patterns like radiation treatment with outcomes. As our study shows preliminary findings on the differences between HPV-positive and HPV-negative HNSCC, we hope that this serves as a basis to investigate the association between HNSCC tumor microenvironment, response to radiation and systemic therapy, and patient outcomes.

## Conclusion

Our study has shown that certain microbes present in tumor biopsy specimens were statistically associated with improved overall survival in patients with head and neck squamous cell carcinoma (HNSCC). These findings suggest that the composition of an individual’s tumor microenvironment can be used to predict patient outcomes, response to treatment, and potentially guide personalized treatment approaches. This is particularly important in the context of HNSCC, as current treatment options can often be associated with significant side effects and may not be effective for all patients. We also found an abundance of pro-tumorigenic M2 polarized macrophages in HPV-negative tumors and increased infiltration of T-cells in HPV-positive tumors. This may help identify mechanism of resistance to immunotherapies and tailor novel immunotherapy combinations in specific patient subgroups. There is still much to understand about the interplay between the microbiome and its reciprocal effects on local gene expression and the immune microenvironment. This study supports further analysis of the microbiome of HNSCC tumors to identify the presence of subsite-specific microbes and to identify targeted therapies that are more likely to be effective in specific patient subgroups. With further prospective research and external validation, these findings have the potential to significantly impact the way we treat HNSCC in the future.

## Supplementary Material

Table S3.xlsx

Table S2.xlsx

Table S5.xlsx

Table S4.xlsx

suppFig2_volcano_differential_gene_expression.png

suppFig1_exorien_forestplot.png

Table S6.xlsx

Table S1.xlsx

suppFig3_hallmark_network_analysis.png

## Data Availability

The raw RNAseq The Cancer Genome Atlas data were accessed from dbGAP. Microbe counts and the scripts to regenerate all analyses and figures are available from https://github.com/spakowiczlab/exohnscHPV.
