# Supplementary figures and images for: Comparative analysis of the tumor microbiome, molecular profiles, and immune cell abundances by HPV status in mucosal head and neck cancers and their impact on survival
Source: Cancer Biol Ther. 2024 May 9;25(1):2350249. doi: 10.1080/15384047.2024.2350249 (PMC11086009; doi:10.1080/15384047.2024.2350249)

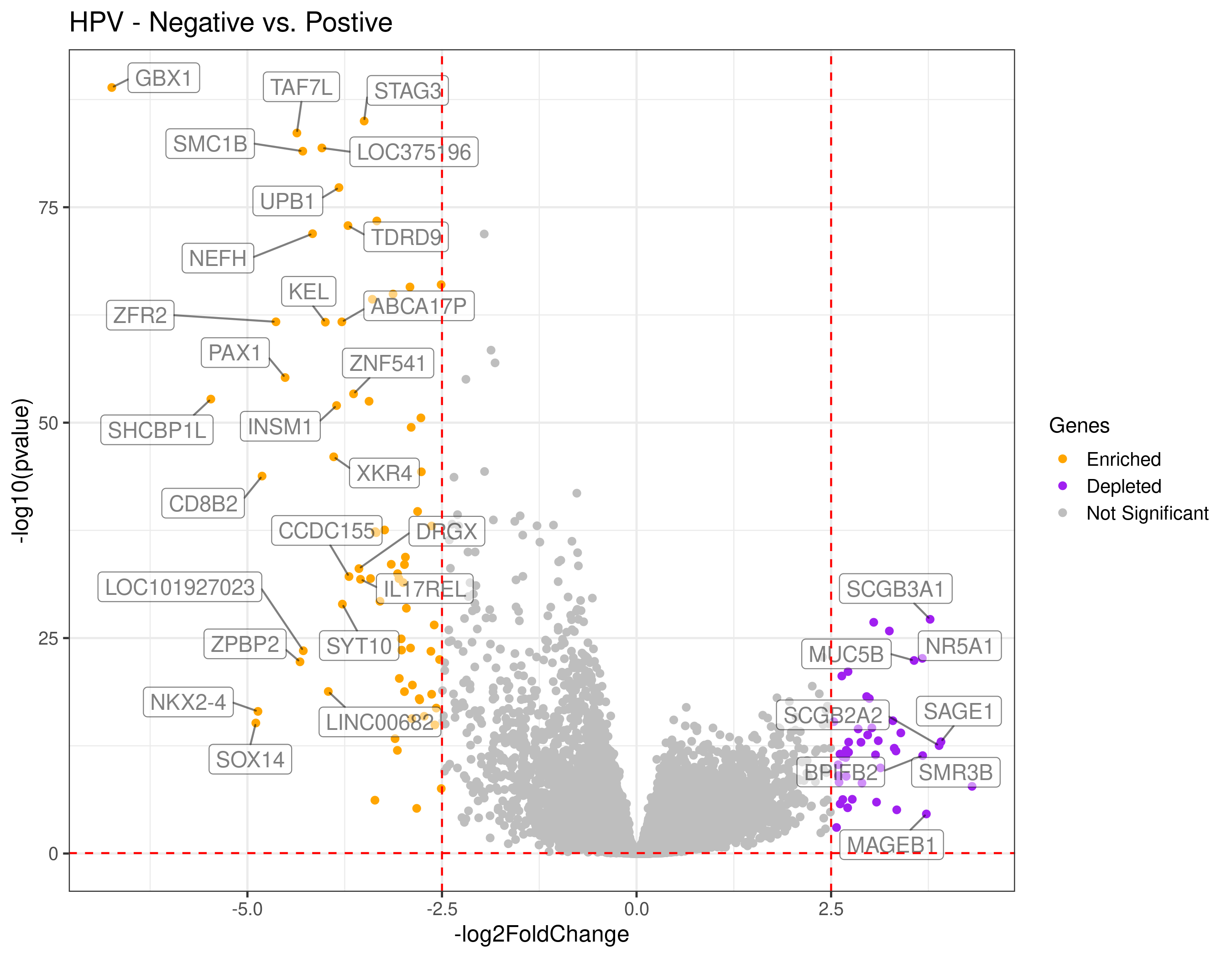

Supplement: suppFig2_volcano_differential_gene_expression.png [file KCBT_A_2350249_SM9684.png]

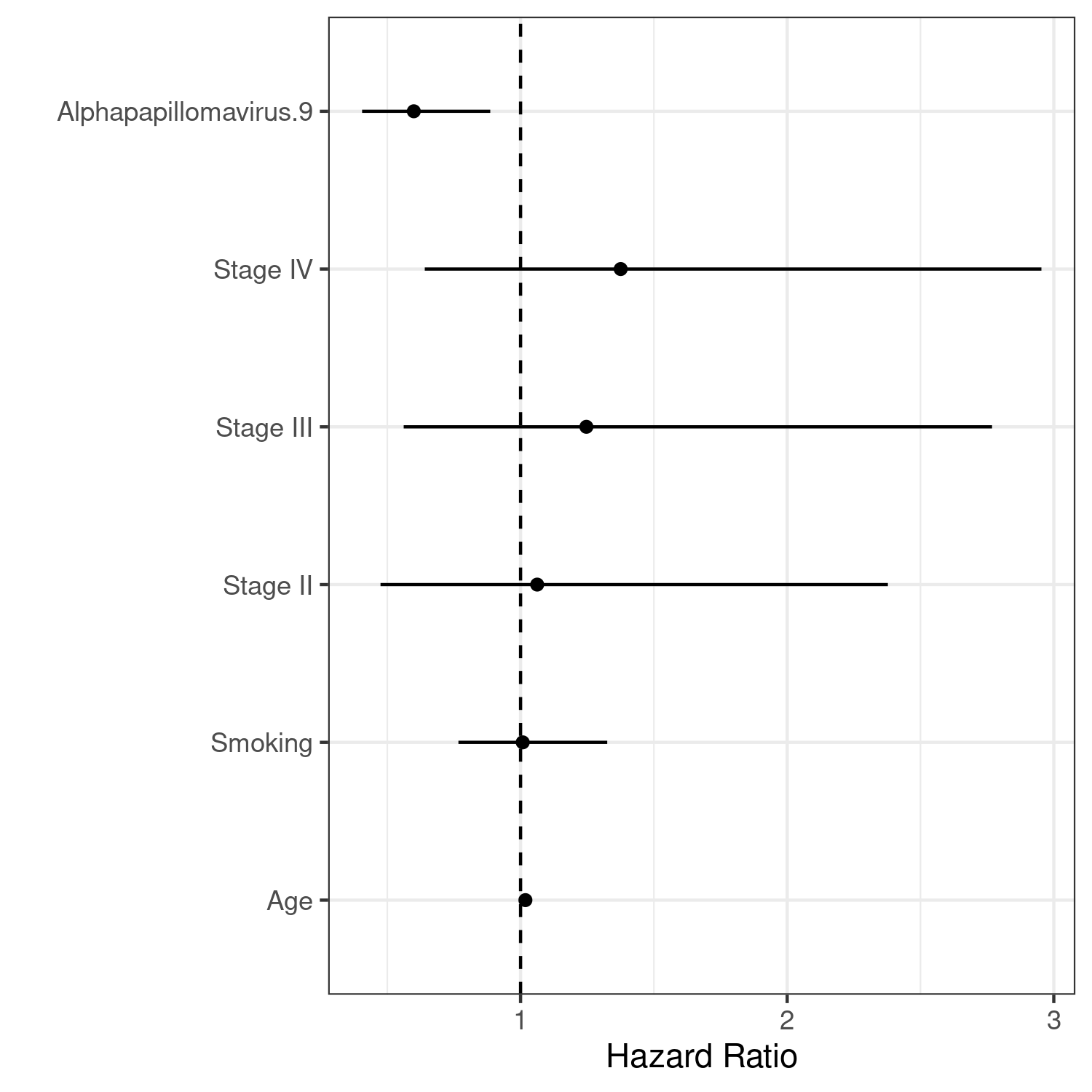

Supplement: suppFig1_exorien_forestplot.png [file KCBT_A_2350249_SM9683.png]

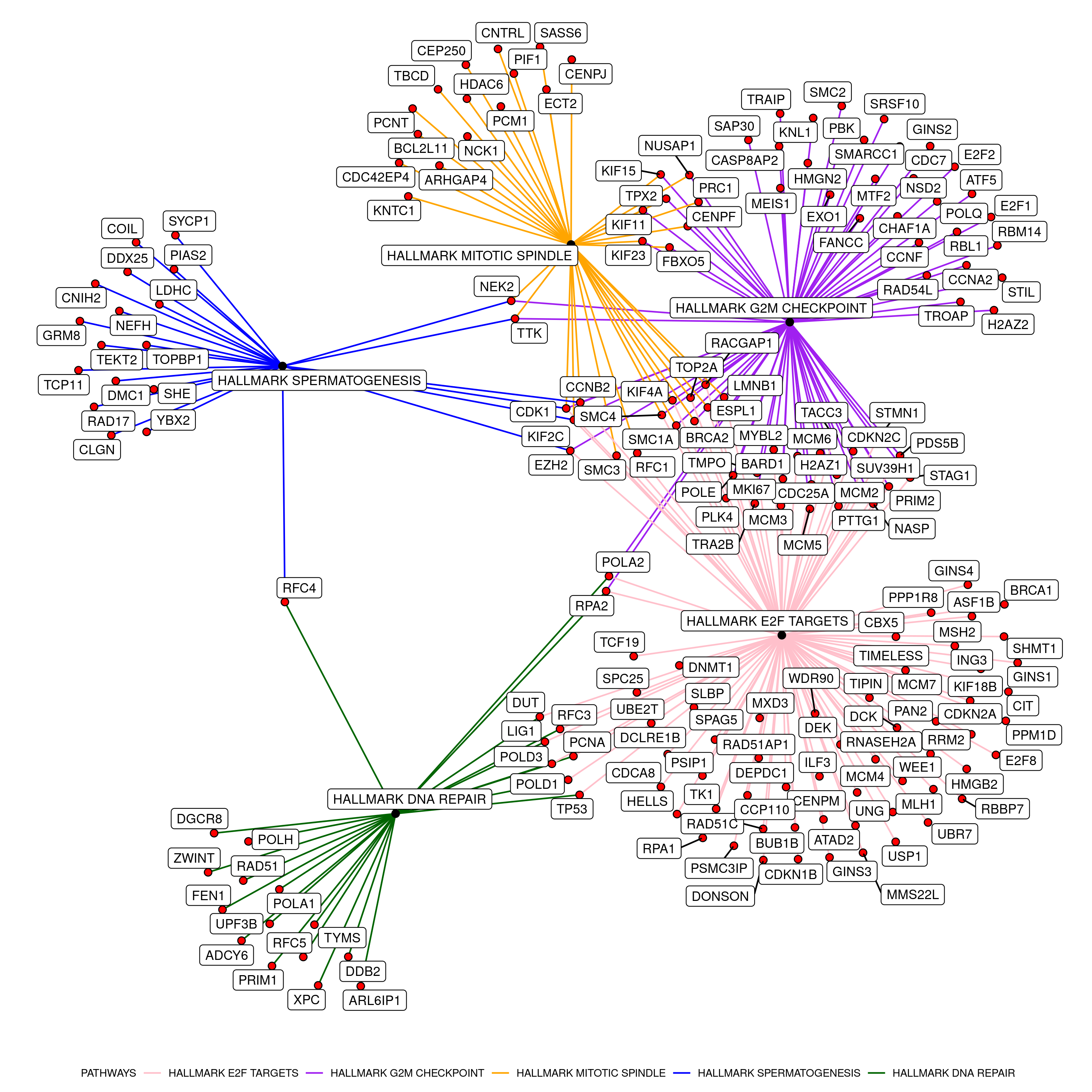

Supplement: suppFig3_hallmark_network_analysis.png [file KCBT_A_2350249_SM9680.png]
